# T201. THE STUDY OF WHITE MATTER MATURATION IN THREE POPULATIONS OF GENETIC HIGH RISK FOR SCHIZOPHRENIA INDIVIDUALS SPANNING THE DEVELOPMENTAL TIMELINE

**DOI:** 10.1093/schbul/sby016.477

**Published:** 2018-04-01

**Authors:** Amanda Lyall, Nathaniel Somes, Fan Zhang, James Robertson, Lauren J O’Donnell, Yogesh Rathi, Ofer Pasternak, Peter Savadjiev, Martin Styner, Zachary Fitzgerald, Raquelle Mesholam-Gately, Heidi Thermenos, Susan Whitfield-Gabrieli, Matcheri Keshavan, Lynn DeLisi, John Gilmore, Larry J Seidman, Marek Kubicki

**Affiliations:** 1 Brigham and Women’s Hospital, Harvard Medical School; 2 University of North Carolina; 3 Beth Israel Deaconess Medical Center, Harvard Medical School; 4 Massachusetts Mental Health Care Center, Harvard Medical School; 5 Massachusetts Institute of Technology; 6 VA Boston Healthcare System

## Abstract

**Background:**

While the etiology of schizophrenia (SZ) is still unclear, it has been characterized as a neurodevelopmental disorder because patients exhibit deviations from normal maturational trajectories that are evident prior to the onset of psychotic symptoms. White matter (WM) has been purported to play a central role in the development of SZ, however, the timing and nature of WM changes in SZ is still poorly understood. This study uses diffusion imaging from three independent Genetic High Risk (GHR) populations spanning the developmental timeline from infancy to young adulthood. The aim of this study is to understand the extent and the time-course of WM maturational pathologies as a function of age and genetic risk for psychosis.

**Methods:**

Two datasets of 3T diffusion-weighted images of children aged 7 to 12 (24 HC and 16 at GHR) and young adults aged 19 to 29 (26 HC and 43 GHR) were collected at the Massachusetts Institute of Technology. The third dataset of 3T images of infants aged 2 years (35 HC and 18 GHR) was collected at the University of North Carolina – Chapel Hill. Whole brain two-tensor tractography was performed and 4 bilateral WM tracts (arcuate fasciculus (AF); inferior longitudinal fasciculus (ILF); cingulum bundle (CB); superior longitudinal fasciculus-ii (SLF-ii)), were extracted utilizing an atlas-guided fiber clustering algorithm. The fractional anisotropy of the tissue (FA-t) was obtained. We carried out group comparisons of FA-t between GHR and HCs utilizing Mann-Whitney-U tests and Cohen’s d effect sizes for each WM tract.

**Results:**

Preliminary analyses reveal significant reductions in FAt between GHR and HC in the right CB (p = 0.013) in the child GHR population. This is mirrored by medium to large effect sizes in the bilateral CB in GHR children (CB-left, d = 0.51; CB-right, d = 0.79). Reductions in FAt in the adult GHR population within the right CB was the largest effect observed in the adult analysis (CB-right, d = 0.46). Effect sizes in the bilateral CB were minimal in the infant GHR population (CB-left, d = 0.14, CB-right, d = 0.11). Significant decreases were also seen in the right SLF-ii in the adult GHR population (p = 0.012), but not in the infant or child GHR populations, though the reductions in FAt in the child GHR population exhibited a small effect (d = 0.35). All other white matter tracts in the adult analysis showed minor effects ranging from d = 0.033 (ILF-right) to 0.28 (ILF-left). The children and infant population also exhibited small effect sizes for all other tracts, with the child GHR dataset ranging from 0.036 (ILF-left) to 0.41 (ILF-right) and the infant GHR dataset ranging from d = 0.038 (SLF-left) to 0.34 (ILF-left).

**Discussion:**

Our preliminary results suggest that abnormal WM maturation may occur in the right CB and right SLF-ii in individuals with increased genetic risk for SZ, specifically after early childhood (7 to 12 years) and into adulthood (19 to 29 years). The CB and SLF-ii are highly implicated in working memory performance, an ability that retrospective studies have shown begins to decline during the peripubertal period in those that develop SZ (~7 to 9 years). The lack of structural findings in GHR infants, may suggest that WM alterations are more likely to arise later in development, thereby possibly identifying childhood as a vulnerable period. Taken together, the preliminary results of this study provide possible evidence of subtle divergences from a healthy WM maturational trajectory in the right CB and right SLF-ii in early to late childhood that may persist into adulthood and these deviations may contribute to cognitive phenotypes described in other studies.

